# Human HemK2/KMT9/N6AMT1 is an active protein methyltransferase, but does not act on DNA in vitro, in the presence of Trm112

**DOI:** 10.1038/s41421-019-0119-5

**Published:** 2019-09-10

**Authors:** Clayton B. Woodcock, Dan Yu, Xing Zhang, Xiaodong Cheng

**Affiliations:** Department of Epigenetics and Molecular Carcinogenesis, University of Texas MD Anderson Cancer Center, Houston, TX 77030 USA

**Keywords:** DNA methylation, Histone post-translational modifications

Dear Editor,

Ever since the initial discovery and characterization of *S*-adenosyl-l-methionine (SAM)^1^, the ATP-activated form of methionine, SAM has become the second most commonly used enzyme cofactor after ATP. A wide variety of macromolecules, including DNA, RNA, proteins, polysaccharides, lipids, and a range of small molecules are subject to methylation by highly specific SAM-dependent methyltransferases (MTases) acting on a particular target atom. Examples of methylation targets include nucleic acids (cytosine-C5, cytosine-N4 and adenine-N6), protein residues (arginine-N, lysine-N, glutamine-N and histidine-N), and small molecules (catechol-O, histamine-N, glycine-N, and thiopurine-S).

Early sequence alignments of known SAM-dependent MTases identified a set of conserved sequence motifs among DNA cytosine-C5 MTases, DNA adenine-N6 and cytosine-N4 MTases, and protein or small molecule MTases^2^. In essence, the two most conserved sequence motifs reflect the common functions of the MTases: motif I (FxGxG) for binding of the methyl donor SAM and motif IV for catalysis. The residues of motif IV conform in accordance with the target atom, for instance, the PC motif (proline-cysteine) is responsible for ring carbon-C5 methylation of cytosine, whereas the (D/N)PP(Y/F/W) motif for exocyclic amino methylation of adenine and cytosine. However, the apparent sequence and structural similarity do not reveal with certainty whether an enzyme acts on a particular substrate (DNA, RNA or protein). Examples include mammalian DNMT2, which shares sequence and structural similarity with DNA cytosine-C5 MTases^3^, but is a tRNA cytosine-C5 MTase;^4^ and *Escherichia coli* (*E. coli*) HemK, which was thought to be a DNA amino MTase^5^, yet is a protein glutamine MTase^6,7^. The conservation in an enzyme’s sequence and structure merely reflects the conserved nature of catalyzing the methyl transfer onto cytosine-C5 (of DNA and RNA) or amino-group (of adenine and glutamine).

Recently, mammalian HemK2 (NCBI Reference Sequence NP_037372.4) has been documented to be a DNA adenine-N6 MTase^8^ (renamed as N6AMT1) and a histone H4 lysine-12 MTase^9^ (renamed as KMT9), in addition to its known activity of glutamine methylation of eukaryotic release factor eRF1^10^. The common feature of the three potential substrates is the amino group (NH_2_) of glutamine, adenine, and lysine (Fig. 1a). It is rare to have a MTase (or any enzyme *per se*) capable of acting on both oligonucleotides and protein (and on two different amino acids). Because previous studies^8,9^ did not compare directly the three potential substrates, here we analyze the in vitro activities of HemK2 on the three substrates side-by-side. HemK2 forms a heterodimeric complex with Trm112^10^ (named after tRNA methylation protein), and we purified recombinant human HemK2-Trm112 heterodimer expressed in *E. coli* (Fig. 1b). Sequence of one of the characterized DNA substrates (GAGTC) of HemK2^8^ overlaps with the recognition sequences (GAnTC) of a known bacterial DNA adenine MTase, *Caulobacter crescentus* cell cycle-regulated MTase^11^, we thus included CcrM as a positive control. In addition, we included *E. coli* Dam (DNA adenine MTase) on GATC for comparison^12^. Both bacterial enzymes were purified in house (Fig. 1b).

**Fig. 1:**
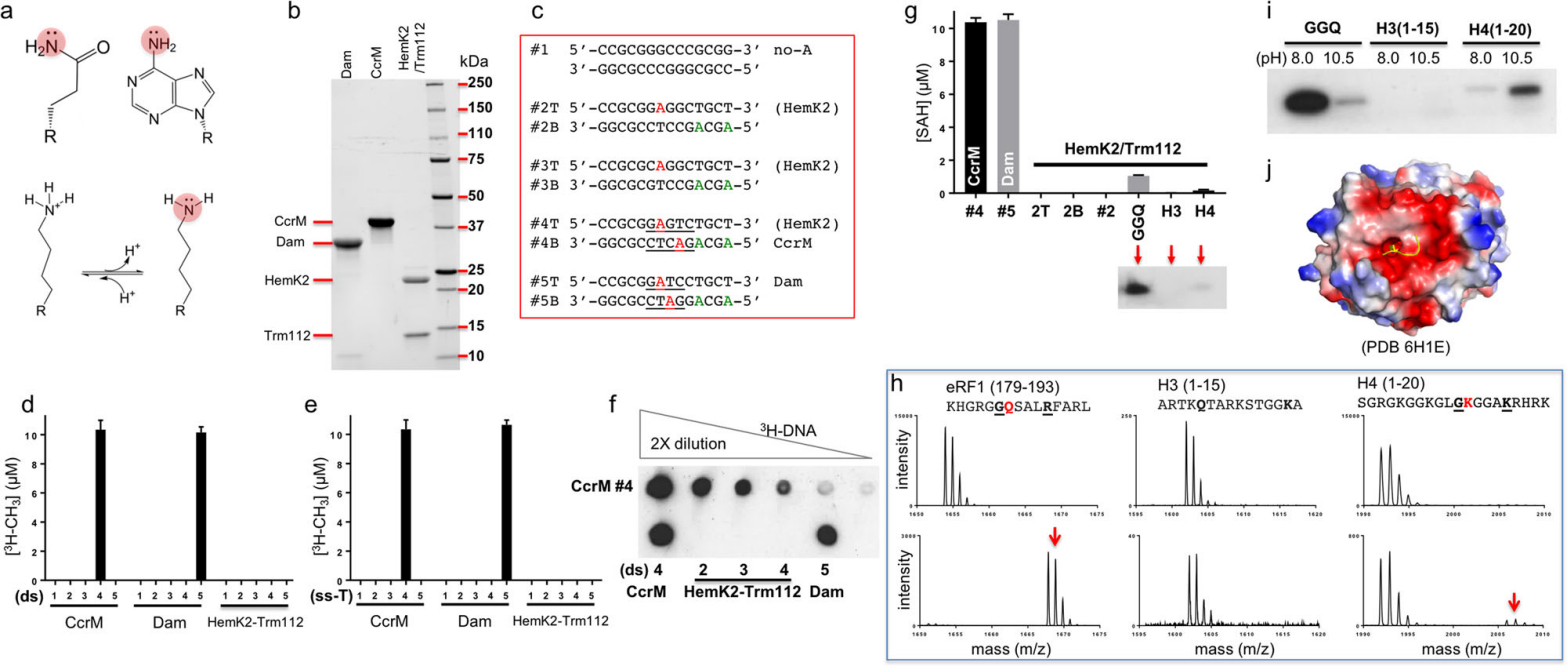
Human HemK2-Trm112 complex methylates peptide substrates. **a** Chemical structures of glutamine, adenine, and lysine. The target nitrogen atom is highlighted in red circle. **b**, **c** Recombinant proteins (panel **b**) and oligonucleotides (panel **c**) used in the study. **d**, **e** HemK2-Trm112 is not active on double-stranded DNA (panel **d**) or single-stranded DNA (panel **e**). **f** Fluorography of reaction products of ^3^H-DNA by indicated enzymes (bottom) and 2 × dilution of oligo #4 by CcrM (top). **g** HemK2-Trm112 is active on GGQ peptide substrates. The vertical axis indicates the reaction by-product (SAH) concentration, measured by the Promega MTase-GloTM assay. Autoradiography films of samples from reactions are shown in the panels below. **h** Representative spectra of MALDI-TOF before (top panels) and after reactions (bottom panels). **i** Effect of pH on the methylation of glutamine-containing and lysine-containing peptides shown by in-gel fluorography. **j** Surface representation of HemK2-Trm112 (PDB 6H1E) shows the substrate binding interface is enriched in negatively charged potential (red: negative; blue: positive, white: neutral). The H4 peptide is shown in yellow with the target lysine inserted into the active site where SAM binds

We designed five short DNA double-stranded oligos (#1 to #5): #1 contains no-adenine and was used as a negative control, #2, #3 and #4 oligos are thought to be substrates of HemK2^8^, #4 contains the CcrM recognition sequence GAnTC and #5 contains the *E. coli* Dam recognition sequence GATC (Fig. 1c—the letters T and B designate the top and the bottom strand respectively). Under the conditions where both CcrM and Dam completed reactions on their respective substrates (CcrM on oligo #4 and Dam on oligo #5), we observed no activities of HemK2-Trm112 on the five oligos examined (either double or single stranded) (Fig. 1d–f). However, we did observe activity of HemK2-Trm112 on peptide derived from eRF1 (residues 179–193), much-reduced activity on histone H4 (residues 1–20), and no activity on histone H3 peptide (residues 1–15) at the condition of pH 8 (Fig. 1g). Under the same condition of pH 8, mass spectrometry analysis of peptide substrates confirmed that glutamine-containing eRF1 peptides were fully methylated, whereas H4 peptides had residual methylated species and no methylation of H3 peptides (Fig. 1h).

Most (if not all) SAM-dependent MTases use the classic S_N_2 reaction mechanism, which requires the target nitrogen be in a deprotonated state. Under the condition (pH 8.0) that HemK2-Trm112 demonstrated strong activity for the glutamine-containing peptide, we observed minor activity on the lysine-containing H4 peptide (Fig. 1i). We reasoned that higher pH conditions might enhance methylation of lysine substrate (with typical pKa value of ~10), as was shown for other biochemically characterized protein (histone) lysine MTases to have optimal in vitro activity at approximately pH 10^13^. After adjusting the pH to 10.5, the activity of HemK2-Trm112 is significantly increased on H4 peptide (Fig. 1i). An investigation employing peptide array libraries showed that HemK2 methylation activity on eRF1 peptide requires a glycine immediately before and an arginine positioned four-residues after the target glutamine^14^. H4 K12 meets this requirement with glycine (G11) and a positively charged lysine (K16) in the place of the corresponding arginine of eRF1 (see sequences shown in Fig. 1h). The short H3 peptide (residues 1–15) used in the assay does contain one glutamine and three lysine residues but is not a methylation substrate of HemK2. Finally, HemK2-Trm112 complex structure (PDB 6H1E^9^) illustrates a negatively charged substrate interface (Fig. 1j), ideal for positively charged peptides such as eRF1 and histone H4, but unsuitable for a negatively charged DNA substrate. We note that the activity on glutamine-containing eRF1 peptide is significantly reduced at the high pH (Fig. 1i), suggesting that high pH is not optimal for catalysis and/or stability of HemK2-Trm112 complex. For the lysine-containing H4 peptide, however, deprotonation of lysine by high pH is preferred for methylation to occur, but only to a much-reduced level as compared with that of glutamine methylation at pH 8.

In sum, using two established MTase assays in vitro, following either incorporation of tritium from ^3^H-SAM into a substrate or formation of byproduct SAH in a bioluminescence assay, we show that human HemK2-Trm112 heterodimer is only active on protein glutamine and lysine, but not active on DNA. In light of current interests on N6-methyladenine in eukaryotic genomic DNA, our study resolves one controversy in the field. Additional data will be required to address the dual specificity of HemK2 on protein glutamine and lysine—a unique property for a MTase working on two different substrates with different target residues, one is located in the cytoplasm (eRF1, glutamine) and the other in nucleus (histone H4, lysine).

Finally, we want to clearly highlight the differences between our studies presented here and that of Xiao et al.^8^. (1) We used recombinant HemK2-Trm112 complex expressed in *E. coli* and purified using a four-column chromatography, whereas Xiao et al. used N6AMT1-Flag plasmid transfected into HEK293T cells and protein purified according to the kit instructions, presumably one-step affinity purification. (2) We used purified HemK2-Trm112 complex (Fig. 1b), whereas there was no mention whether the protein preparation used by Xiao et al. contains Trm112. In a separate study, partially purified Flag-N6AMT1 from overexpressing HEK293T cells appeared to contain a ~15-kDa protein that might be co-purified endogenous Trm112, and this protein preparation did not methylate DNA oligonucleotides in vitro as shown by N6mA specific antibody (see Fig. S4 of Xie et al.^15^). (3) Our fluorography assays on DNA (Fig. 1f) were conducted with [E] = 2 μM, [S] = 10 μM, and [^3^H-SAM] = 10 μM, whereas Xiao et al. used 25 µl reaction system containing [E] = 0.8 μg (~0.8 μM), [S_DNA_] = 250 pmol (=10 µM), [^3^H-SAM] = 55 μM (which is puzzling as that is the concentration of the PerkinElmer NET155V [^3^H-SAM] stock). Both reactions were carried out at 25 °C overnight under similar buffer and salt conditions.

## Supplementary information

Supplementary Information.

## Supplementary information

**Supplementary Information** accompanies the paper at (10.1038/s41421-019-0119-5).
