# Supplementary material for: Human HemK2/KMT9/N6AMT1 is an active protein methyltransferase, but does not act on DNA in vitro, in the presence of Trm112
Source: Cell Discov. 2019 Sep 10;5:50. doi: 10.1038/s41421-019-0119-5 (PMC6796829; doi:10.1038/s41421-019-0119-5)
Supplement: Supplementary file 1 — Supplementary Information. [file 41421_2019_119_MOESM1_ESM.pdf]

Supplementary Information

Woodcock et al.

Human HemK2/KMT9/N6AMT1 is an active protein methyltransferase, but does not act on DNA in vitro, in the presence of Trm112

Clayton B. Woodcock, Dan Yu, Xing Zhang*, Xiaodong Cheng*

Department of Epigenetics and Molecular Carcinogenesis, University of Texas MD Anderson Cancer Center, Houston, TX 77030, USA

*These authors jointly supervised this work: Xing Zhang, Xiaodong Cheng

* e-mail: xzhang21@mdanderson.org; xcheng5@mdanderson.org

Email addresses of all authors:

CBW (CBWoodcock@mdanderson.org); DY (DYu6@mdanderson.org)

Supplementary Methods

Human HemK2-Trm112. Codon-optimized HemK2 gene (GenScript) was cloned into a modified pET28b vector as an N-terminal 6xHis-SUMO tag (pXC2120) and co-transformed into the BL21(DE3) CodonPlus (Stratagene) strain with pXC2076, a pET22b plasmid containing human Trm112 gene. The HemK2-Trm112 was expressed at 16° C and purified through a four-column chromatography conducted in a BIO-RAD NGC™ system: Ni-column followed with cleavage of His-sumo tag by Ulp1 protease, HiTrap Q-SP tandem columns, and size exclusion chromatography.

Bacterial cultures in LB broth were grown at 37°C until $A_{600\ nm}$ ~ 1, shifted to 16°C, induced with the addition of 0.4 mM isopropyl β-D-1-thiogalactopyranoside (IPTG) and cultured for an additional 16 h. The cells were lysed by sonication in the lysis buffer [20 mM HEPES (pH 8.0), 300 mM NaCl, 10% glycerol, 0.5 mM tris (2-carboxyethyl) phosphine (TCEP) and 1 mM phenylmethylsulfonyl fluoride (PMSF)]. The lysate was clarified by centrifugation at 25,000 rpm for 2 h at 4°C and passed through a 3.1 μm filter (Thermo Scientific Titan3 Filter). The supernatant was loaded onto a 5-mL HisTrap Ni-column (GE Healthcare), which was washed with 60 mL of the lysis buffer with 60 mM imidazole. The His-tagged protein was eluted with an imidazole gradient from 60 mM to 300 mM with the peak maximum at 160 mM. Eluted fractions were pooled, diluted into low pH buffer [20 mM HEPES (pH 6.8), 5% glycerol, and 0.5 mM TCEP] to a final concentration of 50 mM NaCl, and incubated overnight with Ulp1 protease (purified in-house) at 4°C, which removes the 6xHis-SUMO tag and leaves two additional N-terminal residues (His-Met). The cleaved protein was further purified by 5-ml HiTrap Q-SP columns (GE Healthcare) connected in tandem. After the sample was loaded the Q column was removed and the SP column was washed with 50 ml of the low pH buffer followed by a 100 mL linear gradient of NaCl from 50 mM to 1 M at a flow rate of 1 mL/min. HemK2-Trm112 complex was eluted and the pooled fractions were concentrated to approximately 2 mL using Amicon Ultra centrifugal filters (10 kDa MWCO) and loaded onto a Superdex 200 16/60 (GE

Healthcare) column equilibrated with 20 mM HEPES pH 8.0, 300 mM NaCl, 5% glycerol, and 0.5 mM TCEP. The complex protein eluted as a single peak.

Tracking tritiated SAM incorporation by scintillation. Reactions tracking product formation by the incorporation of tritium (^{3}H) were conducted in a reaction buffer (50 mM NaCl, 1 mM DTT, 50 mM HEPES pH 8.0 or 20 mM glycine/NaOH 10.5) using a molar ratio of 1:10 for labeled [methyl-^{3}H]SAM (Perkin Elmer NET155V001MC) to unlabeled SAM. DNA oligonucleotides were synthesized by IDT or Sigma, and peptides were synthesized by GenScript or Biomatik. Reactions were initiated by the addition of substrate ([S]=10 μM) into a reaction mixture of enzyme ([E]=2 μM) and SAM (10 μM) at 37°C for 1 h. 5 μL of reactions were blotted onto Hybond-N$^+$ membrane (GE Healthcare) and immediately submerged into 300 mL of 50 mM monophosphate buffer. The membranes were washed three times for 5 min each on an orbital shaker followed by two 5 min washes with 200 proof ethanol and air-dried. The samples were counted using a PerkinElmer Tri-Carb 3110 TR in DPM mode. Background specific to each wash was subtracted from all data points, and the data was plotted using GraphPad Prism.

Bioluminescence assay (Promega MTase-Glo™). Reactions were carried out in duplicates under the same conditions described above and sampled processed according to the manufacturer's instructions. Reactions were terminated by addition of TFA to a final concentration of 0.1% (v/v) and a 5-µl mixture was transferred to a low-volume 384-well plate. The activity was measured using a Promega bioluminescence assay (MTase-Glo) in which the reaction by-product (SAH) is converted into ATP (by a two-step reaction) that is detected through a luciferase reaction. A Synergy 4 Multi-Mode Microplate Reader (BioTek) was used to

measure the luminescence signal.

Reaction products visualized by fluorography. Reactions visualized by film were conducted under the same conditions as the scintillation method described above, except that the reactions were carried out at room temperature (~22 °C) overnight using only [methyl-^{3}H]SAM (without cold SAM). For nucleic acid substrates, an aliquot of 2 µl of reaction mixtures (or dilutions) were blotted directly on to an Hybond-N$^+$ membrane, washed for 5 minutes three times, then air dried without ethanol. We found this method to be more reliable and considerably more sensitive than using native page gels. For autoradiography, the spotted membranes were exposed to Hyperfilm ECL (GE Healthcare) for 16 hr at -80°. For fluorography, the membrane was processed with EN^{3}HANCE (PerkinElmer) and air dried, followed by 16 hr exposure at -80°C with the Amersham Hyperfilm ECL. To visualize reactions of HemK2-Trm112 on peptides, 5 µL of the overnight samples were run on an 18% SDS-PAGE gel. The gel was soaked in EN^{3}HANCE solution for 30 min, dried, and exposed to the Hyperfilm for 36 hr at -80°C.

MALDI-TOF mass spectrometry. Reactions of HemK2-Trm112 on peptides were conducted as room temperature (~22 °C) overnight except that no radiolabeled SAM was used ([E]=10 µM, [S]= 5 µM and [SAM]=20 µM). A 2-µL of sample for each time point was quenched and diluted into 68 µL of 0.1% TFA. A sample of 0.5 µL was then mixed with 0.5 µL of Alpha-CHC (Agilent Technologies) from which 0.6 µL of the mixture was spotted onto a stainless steel MALDI plate. The data was collected at the MD Anderson Proteomics core facility using an Applied Biosystems 4700 MALDI-TOF, a total of 5000 shots were collected per sample using a laser intensity setting of 6000.
